# The predictive value of preoperative luteinizing hormone to follicle stimulating hormone ratio for ovulation abnormalities recovery after laparoscopic sleeve gastrectomy: A prospective cohort study

**DOI:** 10.3389/fendo.2022.1043173

**Published:** 2023-01-05

**Authors:** Fashun Liu, Yue Li, Zhenxiong Ye, Xiaohua Jiang, Ruichen Liu, Zhen Li, Chiye Ma

**Affiliations:** ^1^ Department of General Surgery, Yangpu Hospital, Tongji University School of Medicine, Shanghai, China; ^2^ Department of General Surgery, East Hospital, Tongji University School of Medicine, Shanghai, China; ^3^ Binhai College, Nankai University, Tianjin, China

**Keywords:** luteinizing hormone to follicle stimulating hormone ratio, ovulation abnormalities, laparoscopic sleeve gastrectomy, obesity, sex hormones, fertility

## Abstract

**Introduction:**

Obesity-related ovulation abnormalities (OA) affect fertility. LSG is the most frequent bariatric operation. However, no research has identified a reliable indicator for predicting OA recovery after LSG. The purpose of this research was to examine the prognostic usefulness of preoperative the luteinizing hormone (LH) to follicle-stimulating hormone (FSH) ratio (LFR).

**Methods:**

Our department conducted a prospective study from 2016 to 2021. Venous blood was typically tested 3 days before surgery to get the preoperative LFR. Descriptive data, preoperative and postoperative variables were also collected. Binary logistic regression related preoperative LFR with OA recovery. The receiver operating characteristic (ROC) curve evulated preoperative LFR’s predictive capability.

**Results:**

A total of 157 women with a complete follow-up of one year were included. LFR was the only factor linked with OA (P < 0.001). AUC (area under the ROC curve) = 0.915, cutoff = 1.782, sensitivity = 0.93, and specificity = 0.82.

**Discussion:**

Overall, LSG has a favorable surgical result, with a %TWL of 66.082 ± 12.012 at 12 months postoperatively. Preoperative sexual hormone levels, as expressed by LFR, has the potential to predict the fate of OA following LSG at one year post-operatively.

## 1 Introduction

Obesity is a metabolic illness caused by the accumulation of excess lipids in the body, with a BMI of 30 kg/m2 or more (1). Currently, the incidence is growing internationally, with the prevalence doubling in more than 70 nations between 1980 and 2015, and continues to rise in the majority of countries (2). Moreover, it is of concern that the level of overweight among women of maternal age is more than 20% (3).

Obesity negatively affects female fertility and is associated with infertility, abnormal menstruation (amenorrhea, scanty menstruation, excessive menstruation), ovulation abnormalities (OA), polycystic ovary syndrome (PCOS), miscarriage, sexual dysfunction, pregnancy complications, decreased oocyte quality, reduced uterine tolerance, and neonatal congenital disabilities (4–6). Studies have shown that the risk of infertility in obese women is three times higher than the normal (7). The probability of natural pregnancy decreases linearly with increasing BMI after a BMI ≥ 29 kg/m2, with a 4% decrease for every one kg/m2 increase in BMI (8). On the other hand, a 5% weight loss significantly improves hormone endocrinology and metabolism, such as reducing free testosterone and insulin, increasing FSH and LH, which in turn increases ovulation frequency, adjusts menstrual cycles, increases the chance of spontaneous ovulation and conception, and improves fertility (9).

OA is one of the common complications of obesity and is the main cause of female infertility (10). It can be classified into three groups according to its etiology: 1. hypothalamic insufficiency; 2. Hypothalamus-pituitary-ovarian (HPO) axis dysfunction; 3. ovarian insufficiency (11). Among them, HPO axis dysfunction (group 2) accounts for 85% of OA (11). As key components of the HPO axis, LH and FSH are gonadotropins synthesized and secreted by the anterior pituitary page (12). FSH stimulates the growth and maturation of immature oocytes into mature secondary follicles before ovulation (13), while LH is responsible for inducing ovulation, preparing the fertilized egg for uterine implantation, and producing luteinizing hormone in the ovary by stimulating follicular membrane cells and luteinized granulosa cells (14). Disorders of LH and FSH will inhibit follicular maturation, cause follicular atresia, and affect follicular cell recruitment and ovulation, leading to sporadic ovulation or anovulation, thus reducing fertility (15). Actually, certain components of the blood, such as inflammatory cell count (16), serum (17), and exosomes are associated with disease progression or surgical prognosis (18). LFR is associated with PCOS (15), visceral adipose dysfunction (19), live birth rate after embryo transfer (20), vascular endothelial growth factor levels (21), and oocytes’ number and competence (22), suggesting that LFR may be a potential disease predictive markers.

In the early 2000s, Professor David Johnston first used a longer, small curvature vertical gastric tube to perform a more physiologic form of gastroplasty known as sleeve gastrectomy (23). Currently, LSG is the most commonly performed bariatric procedure in the United States and worldwide (24). Long-term follow-up studies have shown that LSG significantly increases postoperative patient pregnancy, live birth, and vaginal delivery rates and reduces pregnancy complications compared to the group without surgical treatment (25). However, no study has yet proposed a prognostic marker for the recovery of OA after LSG. Considering the relationship between LH, FSH, and ovulation, we evaluated the correlation between preoperative LFR and postoperative clinical outcomes. We hypothesized that the level of LFR could be prognostic for OA recovery after LSG.

## 2 Materials and methods

### 2.1 Participants

A prospective consecutive cohort of patients who underwent LSG at weight reduction and metabolic clinic between January 2016 and January 2021 were included. The research has gained approval from the local ethics committees. The subjects’ anonymity and confidentiality were under the Helsinki Declaration. The inclusion criteria for the cohort were as follows:

1. Women aged 14 to 45;
2. Met the indications for bariatric surgery;
3. Preoperatively diagnosed with OA.
4. The exclusion criteria were as follows:
5. Serious surgical complications;
6. Failure to follow up as scheduled;
7. Inability to comply with postoperative dietary and lifestyle modifications;
8. Drug or alcohol addiction or unmanageable mental disease;
9. Intellectual disability or immaturity, inability to regulate own behavior;
10. Recent use of hormonal contraceptives or medications that impact ovulation
11. Affected by hypothyroidism or Cushing’s disease.

### 2.2 Source of data and clinical variables

All procedures were performed by a senior bariatric surgeon under primary care. A 36# gastric support tube was used to determine the extent of resection. One senior bariatric surgeon (C.Y.M.) was in charge of tallying the data. The preoperative data collection comprised demographic information and menstrual history. In addition, venous blood samples were collected from fasting subjects in order to determine levels of fasting glucose, glycosylated hemoglobin, fasting insulin, fasting C-peptide, uric acid, LH, FSH, testosterone (TESTO), sex hormone-binding globulin (SHBG), estradiol (E2), total cholesterol (TC), low-density lipoprotein (LDL), high-density lipoprotein (HDL), and triglyceride (TG). Anthropometric indicators, follicular phase sex hormone levels, and ovulation data were also gathered at 3, 6, and 12 months after surgery, respectively. The following calculations were performed during our investigation: (1) LFR defined as LH content (mIU/mL) divided by FSH content (mIU/mL) measured three days before the operation; (2) BMI was calculated by dividing the weight in kilograms by the square of the height in meters; (3) % TWL was determined by multiplying (reduced body mass/preoperative body mass) by 100%; (4) Following was the formula for calculating HOMA-IR: glucose concentration during fasting * insulin concentration during fasting/22.5; (5) Excess BMI (EBMI) was equaled to current BMI minus standard BMI (25 kg/m^2^); when current BMI was less than 25 kg/m^2^, EBMI was 0.

### 2.3 Statistical examination

The descriptive analysis of baseline subject characteristics included continuous variables represented as means plus or minus standard deviations and categorical variables as frequencies (percent). The distribution of the data contained in this research was examined to ensure that it roughly satisfies the normality and chi-square tests. Paired-samples T-tests were used to compare the changes in each indicator before and after the intervention for each participant. Binary logistic regression was performed to analyze the factors associated with the recovery of ovulatory capacity at 12 months postoperatively, corrected for the relevant factors (age, BMI). ROC curves were constructed to evaluate the indicators’ predictive potential, and the Jorden index was utilized to calculate the predictive threshold. Sample size estimation was performed using MedCalc software (version 20.0.3) software with specific settings of α:0.05,1-β:0.9 and null hypothesis of AUC=0.8. This study’s statistical analyses were conducted using SPSS 26.0, and P < 0.05 was deemed statistically significant.

## 3 Results

### 3.1 Population characteristics

In all, 181 patients met the inclusion criteria: 157 patients (86.74%) were successfully followed up for 12 months. **Table 1** summarizes the descriptive features and laboratory findings at baseline for the individuals enrolled in the trial. The preoperative mean values of BMI (36.64 ± 6.366 kg/m2), HOMA-IR (11.81 ± 13.613), UA (394.03 ± 96.455 umol/L), TESTO (45.61 ± 19.584 ng/mL), insulin (32.39 ± 28.360 pmol/L), C-peptide (4.51 ± 1.887 nmol/L), LDL (3.43 ± 0.894 mmol/L) were above the normal range. Moreover, the mean SHBG (25.06 ± 24.287 nmol/L) was below normal. Other indicators did not exhibit substantial abnormalities. 37 patients (23.6%) had diabetes, whereas 22 (17.5%) had hypertension. Notably, PCOS was preoperatively detected in 120 (76.4%) participants. Despite the fact that only 23.6% of persons were diagnosed with diabetes, obese patients needed greater amounts of fasting insulin and C-peptide to maintain normal blood glucose levels. In addition, OA was highly related to PCOS, with over 75% of OA patients in our research suffering from PCOS.

**Table 1:** Preoperative Characteristics of Patients Included^a^.

Characteristics	Values
Age, years	28.8 ± 6.298
High, m	1.64 ± 0.050
Mass, kg	99.04 ± 17.850
BMI, kg/m^2^	36.64 ± 6.366
GLU, mmol/L	6.34 ± 2.1148
HbA1c, %	6.23 ± 1.247
HOMA-IR	11.81 ± 13.613
UA, umol/L	394.03 ± 96.455
LH, mIU/mL	8.75 ± 7.297
FSH, mIU/mL	5.53 ± 2.282
LH/FSH	1.63 ± 0.899
Testosterone, ng/mL	45.61 ± 19.584
Sex Hormone-binding globulin, nmol/l	25.06 ± 24.287
Insulin, pmol/l	32.39 ± 28.360
C-peptide, nmol/l	4.51 ± 1.887
Total cholesterol, mmol/l	5.03 ± 0.969
Low Density Lipoprotein, mmol/l	3.43 ± 0.894
High Density Lipoprotein, mmol/l	1.21 ± 0.286
Triglyceride, mmol/l	2.00 ± 1.665
Diabetes, %	37 (23.6)
Hypertension, %	22 (17.5)
Polycystic ovarian syndrome, %	120 (76.4)

a Data are presented as mean ± SD and frequencies (%).

### 3.2 Postoperative weight and body mass index

**Figure 1:**
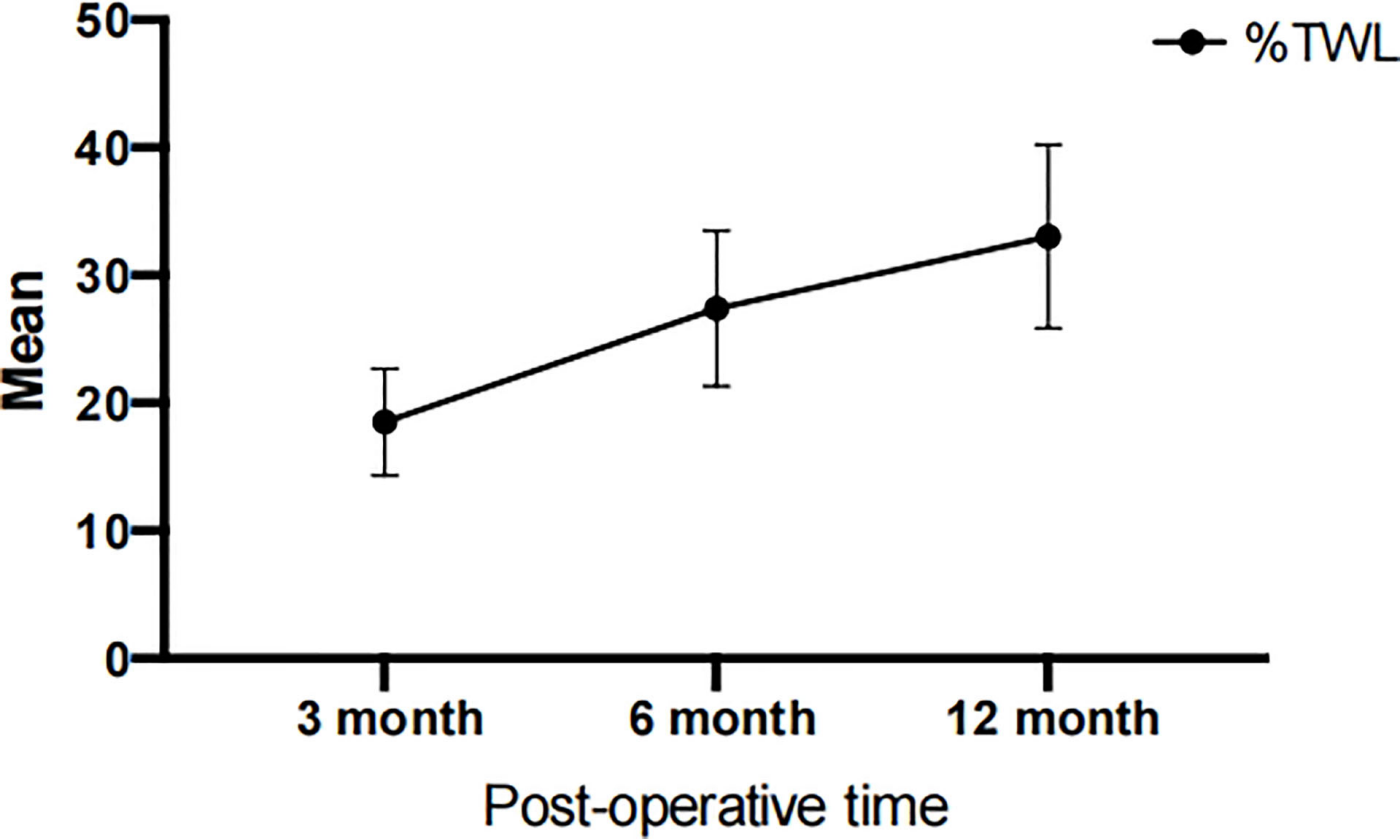
Postoperative %TWL at 3, 6, and 12 months. TWL, Total weight loss.

According to **Table 2**, the average weight of patients before surgery was 99.04 ± 17.850 kg, which was reduced to 81.13 ± 15.31 kg, 71.95 ± 13.34 kg, and 66.08 ± 12.01 kg at 3, 6, and 12 months after surgery, respectively. Meanwhile, the patient’s BMI improved from 36.64 ± 6.37 kg/m^2^ to a normal level of 24.39 ± 4.10 kg/m^2^. Furthermore, the EBMI reduced from 11.64 ± 6.37 kg/m^2^ to 0 kg/m^2^. In the past, the percentage of extra weight loss was considered a measure of the effectiveness of bariatric surgery; however, the current opinion is that %TWL is a superior indicator (26). We evaluated the variations in TWL among the patients. LSG was successful, with a mean % TWL of 33.04 ± 7.19 at 12 months after the procedure. **Figure 1** and **Table 2** demonstrated that patients lost an average of 27.38 ± 6.08% of their total body weight within the first six months post-operatively. At six to twelve months after surgery, individuals continued to lose weight at a slower pace (compared to the first six months after surgery), which implying that people lose the most weight over the first six months after LSG. The first six months after LSG is a prime time for patients to lose weight and may require more attention.

**Table 2:** Comparison of preoperative and postoperative weight indexes at 3, 6 and 12 months^a^.

	Model-based estimates Mean ± SD				P value		
	Baseline	3 months	6 months	12 months	3 months vs baseline	6 months vs baseline	12 months vs baseline
Mass, kg	99.04 ± 17.85	81.13 ± 15.31	71.95 ± 13.34	66.082 ± 12.01	<0.001*	<0.001*	<0.001*
BMI, kg/m^2^	36.64 ± 6.37	30.06 ± 5.44	26.64 ± 4.70	24.39 ± 4.10	<0.001*	<0.001*	<0.001*
EBMI, kg/m^2^	11.64 ± 6.37	5.06 ± 6.37	1.64 ± 6.37	0	<0.001*	<0.001*	<0.001*

^*^The difference reached statistical significance.

a Preoperative and postoperative mass and BMI are expressed as mean ± SD.

### 3.3 Variations in sexual hormone levels

**Figure 2:**
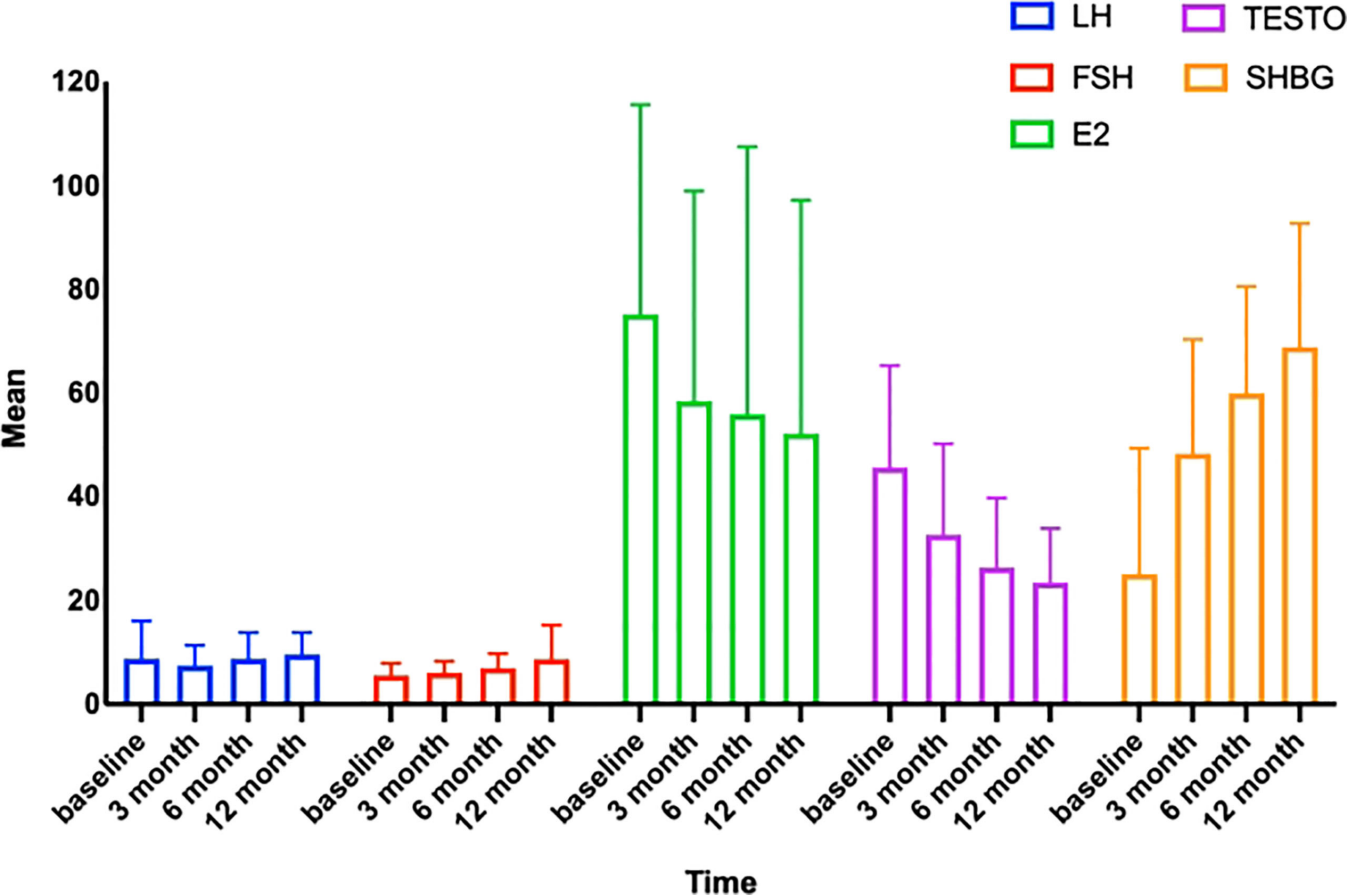
Trends in sex hormones with time. LH, luteinizing hormone; FSH, follicle-stimulating hormone; TESTO, testosterone; SHBG, sex hormone-binding globulin; E2, estradiol.

Since obese people often have altered sex hormone levels leads to reduced fertility. Our subsequent research will investigate if LSG may reverse these detrimental changes. During the follicular period, we collected and statistically examined the levels of LH, FSH, TESTO, E2, and SHBG in the patients’ venous blood before surgery and 3, 6, and 12 months after surgery. At 12 months postoperatively, the patients’ LH rose from 8.75 ± 7.297 to 9.60 ± 4.230 mIU/ml, FSH increased from 5.53 ± 2.282 to 8.70 ± 6.543 mIU/ml, SHBG increased from 25.06 ± 24.287 to 68.86 ± 23.773 nmol/L, E2 fell from 75.15 ± 85.124 to 52.14 ± 44.591 pg/mL, and TESTO decreased from 45.61 ± 19.584 to 23.43 ± 10.345 ng/mL. LH, FSH, and SHBG rose progressively after surgery, while E2 and TESTO exhibited the reverse pattern (**Table 3**, **Figure 2**). At three months postoperatively, variations in sex hormone levels were noticed; the findings were statistically significant, and the pattern of alterations tended to stabilize. In addition, E2, TESTO, and SHBG altered higher, and all index changes stabilized.

**Table 3:** Comparison of preoperative and postoperative sex hormone levels at 3, 6 and 12 months^a^.

	Time				P value		
	Baseline	3 months	6 months	12 months	3 months vs baseline	6 months vs baseline	12 months vs baseline
LH, mIU/mL	8.75 ± 7.297	7.43 ± 3.919	8.76 ± 5.049	9.60 ± 4.230	0.031*	<0.001*	<0.001*
FSH, mIU/mL	5.53 ± 2.282	6.05 ± 2.225	6.97 ± 2.794	8.70 ± 6.543	0.017*	<0.001*	<0.001*
E2, pg/mL	75.15 ± 85.124	58.44 ± 51.111	55.90 ± 61.063	52.14 ± 44.591	0.027*	0.027*	0.028*
TESTO, ng/mL	45.61 ± 19.584	32.62 ± 17.464	26.30 ± 13.302	23.43 ± 10.345	<0.001*	<0.001*	<0.001*
SHBG, nmol/L	25.06 ± 24.287	48.31 ± 21.954	59.93 ± 20.617	68.86 ± 23.773	<0.001*	<0.001*	<0.001*

^*^The difference reached statistical significance.

### 3.4 Correlation between recovery of OA and LFR

**Figure 3:**
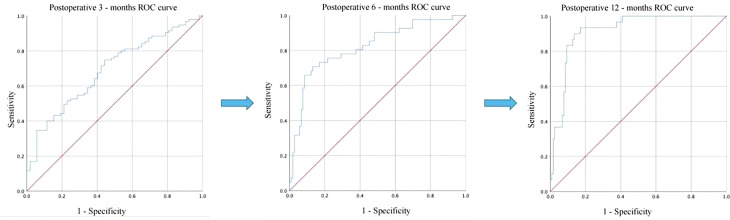
Comparison of the predictive power of LFR for postoperative OA recovery at 3, 6, and 12 months.

To identify acceptable indicators for predicting the resumption of OA in patients after surgery, we first conducted a univariate binary logistic regression. According to **Table 4**, LFR was substantially related to OA at 12 months after surgery (P < 0.001, B = 6.775, 95% CI: 3.377, 13.594). On this premise, a multifactorial binary logistic regression was performed, adjusting for age and BMI, and LFR at baseline was still substantially linked with OA at 12 months (P < 0.001, B = 6.127, 95% CI: 3.022, 12.422) (**Table 5**). The ROC curves at 3, 6, and 12 months were generated to establish the predictive effect of preoperative LFR. The findings are shown in **Figure 3**, where the area under the curve was 0.689, 0.821, and 0.915 at 3, 6, and 12 months postoperatively, demonstrating the predictive capacity of LFR for OA. The area under the curve grew with time, and the predictive ability improved. The greatest predictive power was seen 12 months after surgery when the area under the curve was 0.915 (P < 0.001, 95% CI: 0.867, 0.964). The Uden index peaked when the cut-off value of LFR was 1.782. (**Figure 3**). The specificity was 0.829, and the sensitivity was 0.933.

**Table 4:** Prognostic factors for recovery of OA at 12 months postoperatively.

	B	95% CI	P value
age	0.948	0.886, 1.016	0.129
Mass	0.979	0.954, 1.004	0.105
BMI	0.939	0.873, 1.001	0.094
LH/FSH	6.775	3.377, 13.594	<0.001*
E2	1.003	0.999, 1.007	0.178
TESTO	7.142	0.953, 53.538	0.056
SHBG	0.981	0.929, 1.036	0.489
GLU	0.774	0.571, 1.050	0.100
HbA1c	0.881	0.602, 1.290	0.515
Insulin	0.998	0.984, 1.013	0.816
C-peptit	0.850	0.666, 1.085	0.191
HOMA-IR	0.940	0.868, 1.017	0.121
TC	0.768	0.488, 1.208	0.254
LDL	0.796	0.497, 1.276	0.344
HDL	0.378	0.079, 1.804	0.223
TG	1.002	0.749, 1.341	0.989
UA	0.999	0.994, 1.003	0.511
Diabetes	0.607	0.213, 1.731	0.350
Hypertension	1.360	0.469, 3.946	0.571
PCOS	2.554	0.827, 7.882	0.103

^*^The difference reached statistical significance.

**Table 5:** Corrected for age, BMI for multi-factor binary logistic regression.

	B	95% CI	P value
age	0.975	0.890, 1.069	0.594
BMI	0.982	0.898, 1.075	0.701
LH/FSH	6.127	3.022, 12.422	<0.001

### 3.5 Analysis of correlations between LFR and clinical variables

The 157 patients were categorized according to the LFR cut-off value, and 52 of them had a preoperative LFR > 1.782. **Table 6** provides preoperative data, and the findings indicate that the weight, BMI, HOMA-IR, and TC of these 52 individuals were lower than those with LFR < 1782. However, the frequency of PCOS was substantially greater, and E2 and TESTO levels were significantly higher, indicating that preoperative sex hormone levels were much more disturbed in patients with LFR > 1.782 (**Table 6**). Regarding postoperative markers, the variations in weight and BMI between the two groups were statistically significant at 3 and 6 months, but there was no difference between the two groups at 12 months. Indicates that patients have continuous weight loss independent of OA recovery. TESTO levels were also greater in the LFR > 1.782 group than in the LFR < 1.782 group at six months, but the differences were not statistically significant at 3 and 12 months (**Table 7**). These results show that the existing LFR cut-off setting has useful.

**Table 6:** Association with preoperative indices according to the cut-off value classification of LFR.

	LFR<1.782 (n=105)	LFR≥1.782 (n=52)	P value
High, m	1.64 ± 0.053	1.64 ± 0.046	0.997
Mass, kg	101.22 ± 18.436	94.63 ± 15.871	0.029*
BMI, kg/m2	37.49 ± 6.645	35.04 ± 5.514	0.024*
GLU, mmol/L	6.53 ± 2.291	5.97 ± 1.786	0.121
HbA1c, %	6.29 ± 1.262	6.12 ± 1.222	0.428
Insulin, pmol/L	33.61 ± 32.05	29.91 ± 18.86	0.442
C-peptit, nmol/l	4.67 ± 2.062	4.19 ± 1.436	0.129
HOMA-IR	13.80 ± 16.289	8.49 ± 6.091	0.045*
TC, mmol/L	5.14 ± 1.036	4.82 ± 0.782	0.049*
LDL, mmol/L	3.52 ± 0.962	3.25 ± 0.716	0.071
HDL, mmol/L	1.19 ± 0.292	1.24 ± 0.272	0.310
TG, mmol/L	2.11 ± 1.930	1.80 ± 0.915	0.281
UA, umol/L	398.14 ± 100.126	385.71 ± 88.934	0.449
Diabetes, %	29 (27.6)	8 (15.4)	0.089
Hypertension, %	15 (19.0)	7 (14.9)	0.558
PCOS, %	73 (69.5)	47 (90.4)	0.004*
E2, pg/mL	65.13 ± 79.574	95.40 ± 93.643	0.036*
TESTO, ng/mL	42.21 ± 19.079	52.47 ± 19.147	0.002*
SHBG, nmol/L	26.37 ± 28.544	22.51 ± 12.464	0.461

* The difference reached statistical significance.

**Table 7:** Association with postoperative indicators at 3, 6 and 12 months according to the cut-off value of LFRa.

	LFR<1.782 (n=105)			LFR≥1.782 (n=52)		
Time	3months	6 months	12 months	3 months	6 months	12 months
Mass, kg	83.03 ± 16.1447*	73.87 ± 14.118*	67.25 ± 12.666	77.43 ± 12.894*	68.53 ± 11.160*	63.80 ± 10.376
BMI, kg/m2	30.75 ± 5.698*	27.38 ± 4.94*	24.92 ± 4.255	28.71 ± 4.560*	23.64 ± 3.694*	25.34 ± 3.955
%TWL	18.77 ± 3.927	27.60 ± 5.719	33.67 ± 6.939	17.99 ± 4.627	27.00 ± 6.712	31.81 ± 7.573
Sex Hormone Level						
E2, pg/mL	57.90 ± 56.765	63.44 ± 71.358	55.78 ± 50.740	60.04 ± 31.849	37.89 ± 17.670	41.71 ± 18.889
TESTO, ng/mL	32.95 ± 17.780	23.90 ± 12.443*	22.49 ± 10.624	31.62 ± 17.381	31.90 ± 11.885*	26.12 ± 9.712
SHBG, nmol/L	51.04 ± 23.974	60.89 ± 22.248	68.67 ± 25.452	41.32 ± 13.938	57.87 ± 17.150	69.38 ± 19.435

a Preoperative and postoperative data are expressed as mean ± SD.

* The difference reached statistical significance.

## 4 Discussion

After examining the outcomes of preoperative and postoperative trials, the present investigation d.7iscovered that LFR levels were the sole component linked with OA. Patients with a preoperative LFR of >182, who had a greater incidence of PCOS and more disrupted sex hormone levels, did not seem to regain ovulation despite achieving the same weight reduction 12 months after surgery.

As the incidence of obesity has grown in recent years, more emphasis has been paid to its complications. Obese women of childbearing age often experience infertility. The primary mechanism may include insulin resistance and hyperinsulinemia in the patient, which leads to increased ovarian androgen production and hinders the synthesis of sex SHBG in the liver, hence increasing the concentration of free sex hormones when SHBG levels are low. The increasing number of free sex hormones in the body subsequently acted on the HPO axis, resulting in malfunction of the HPO axis and decreased FSH and LH secretion (27). A 4-year follow-up study of 106 women following bariatric surgery revealed that bariatric surgery improved patients’ sex hormone levels. Comparable results were achieved in our investigation, with E2, LH, FSH, and SHBG much higher and TESTO lower than before surgery, with a fairly steady trend (28).

Additionally, excess adipocytes in the body emit pro-inflammatory mediators including macrophage chemoattractant protein - 1, tumor necrosis factor-α, interleukin- 6, and free fatty acids, which encourage the recruitment and activation of adipose tissue macrophages and the release of pro-inflammatory cytokines (29). At the same time, alterations in the gut microbiota lead to an increase in lipopolysaccharide in the follicular fluid of cystic follicles, which activates downstream pathways *via* Toll-like receptor 4 and activates macrophages, polarizing them toward the pro-inflammatory phenotype Macrophages 1 and secreting pro-inflammatory cytokines and chemokines to cause an increase in the infiltration of monocytes (30). On the basis of these alterations, chronic inflammation of the ovaries in obese women over an extended period of time decreases the number of primordial follicles and increases the number of luminal and atretic follicles, depleting the ovarian reserve and causing infertility.

On the other hand, the endometrial tolerance of obese women is lowered owing to metabolic changes, and the uterine implantation window is shifted due to a higher BMI, which impacts embryo implantation and contributes to an increase in miscarriage and a poor fertility rate (31–33). High embryo quality is essential to the success of assisted reproductive technology; yet, obese women have considerably poorer cell formation, embryo quantity, blastocyst formation rate, pregnancy rate, embryo implantation rate, live birth rate, and miscarriage rate compared to normal women (34). Experiments on animals have shown that maternal obesity causes telomere dysfunction and impairs chromosomal stability, leading in lower oocyte quality and diminished embryonic developmental potential (35).

The processes by which obesity impairs female fertility are complicated and multidimensional, and our work demonstrates that LSG enhances female fertility by modifying sex hormone levels and restoring the normal function of the HPO axis. However, it should be noted that the existence of ovarian inflammation and lower embryo quality remain unaddressed and that further clinical and fundamental trials are required.

LSG has the advantages of simple surgical approach, short learning curve, no alteration of the patient’s original physiological access, fewer complications, and comparable weight loss and resolution of comorbidities to traditional bariatric surgery, making it more popular among bariatric metabolic surgeons and patients (36). LSG may decrease the weight of morbidly obese patients in the short term, improve their menstrual cycle, restore ovulation, and enhance fertility by improving the pregnancy rate, live birth rate, vaginal delivery rate, and effectively reducing the incidence of pregnancy complications (37, 38). Moreover, FSFI ratings were considerably lower in obese than in healthy women, and scores on all components of the FSFI scale rose in LSG patients and recovered to normal by 6 months post-operatively (39). PCOS is a common endocrine illness characterized by monthly irregularities, ovulation abnormalities, polycystic ovaries, and hyperandrogenemia, all of which have a substantial influence on female fertility. Obesity and insulin resistance are the most prevalent risk factors for PCOS (40). Obesity and PCOS have reciprocal effects: obesity can aggravate PCOS symptoms, and PCOS can exacerbate obesity (41). After LSG, patients with obesity and PCOS had decreased free androgens, increased SHBG, and decreased ovarian volume to normal range. The majority of PCOS patients resume a regular menstrual cycle and ovulate on their own, and clinical symptoms such as hirsutism, rough pores, and acne caused by high circulating testosterone levels are alleviated to variable degrees (42).

No research has investigated the variables related to OA recovery after LSG. It is general knowledge that OA is prevalent in obese persons. OA is a major factor contributing to female infertility and is characterized by anovulation. The most common cause of OA is PCOS, showing that 70% of anovulatory women suffer from PCOS. Similarly, our findings reveal a prevalence of 76.4% for PCOS. It is well known that there is a significant correlation between obesity and PCOS, and LSG for PCOS is currently a hot research topic. In the present study, over 100 patients resumed normal ovulation at 12 months postoperatively, which may provide a new potential modality for treating obesity with OA. To better direct the use of LSG in the clinic, it is necessary to identify a reliable marker as a predictive target. After adjusting for confounding factors such as age and BMI, we discovered that LFR might be utilized to predict ovulatory capability after LSG. The ROC curve further establishes the predictive power of the LFR with an area under the curve of 0.915 (P < 0.001, % 95CI: 0.867, 0.964). The area under the ROC curve for LFR at 3 and 6 months was 0.689 and 0.821, indicating that the prognostic ability of LFR for postoperative OA increases with time. LFR is associated with obesity, insulin resistance (15), hyperglycemia (43), and chronic inflammation in PCOS (44). Consequently, our subsequent investigation was stratified by LFR cut-off values, and it was discovered that LFR was correlated with patient weight, BMI, HOMA-IR, TC, PCOS prevalence, E2, and TESTO. These findings show that LFR may be a promising disease prognostic diagnostic, not only for OA.

This research has several drawbacks. Only the correlation between preoperative LFR and postoperative OA recovery was investigated. Considering that LFR values acquired at various periods may fluctuate due to age, food, or other variables during follow-up and that LH and FSH levels are associated with the menstrual cycle, ongoing monitoring is required. In addition, as no previous studies have studied the link between LFR and surgical recovery, sample size estimates could not be performed prior to patient admission. For this purpose, we used MedCalc software (version 20.0.3) (45) for testing and estimated a sample size of 118 cases, which was covered by our cohort with 157 cases. Additional research is required to confirm our results and values. Finally, we followed our patients for one year after surgery. Longer follow-up data is necessary.

## 5 Conclusion

Overall, LSG has a favorable surgical result, with a %TWL of 66.082 ± 12.012 at 12 months postoperatively. Preoperative sexual hormone levels, as expressed by LFR, has the potential to predict the fate of OA following LSG at one year post-operatively.

## Data availability statement

The original contributions presented in the study are included in the article/supplementary material. Further inquiries can be directed to the corresponding authors.

## Ethics statement

The studies involving human participants were reviewed and approved by Ethics Committee Approval of Yangpu Hospital Yangpu Hospital, Tongji University School of Medicine, Shanghai 200090, China. Written informed consent for participation was not required for this study in accordance with the national legislation and the institutional requirements.

## Author contributions

FL and YL contributed equally to this work. Conceptualization, ZL and CM; methodology, YL; software, ZY; validation, ZL, CM, ZY, XJ, and FL; formal analysis, FL; data curation, CM and XJ; writing—original draft preparation, FL; writing—review and editing, CM; visualization, ZY and YL. All authors have read and agreed to the published version of the manuscript.
